# 815. False Negative BioFire FilmArray Meningitis/Encephalitis Multiplex PCR Assay in Cryptococcal meningitis: A Single Center Analysis

**DOI:** 10.1093/ofid/ofad500.860

**Published:** 2023-11-27

**Authors:** Nancy Dang-Orita, Alec M Chan-Golston, Marilyn Mitchell, Geetha Sivasubramanian

**Affiliations:** University of California San Francisco- Fresno, Fresno, California; School of Social Sciences, Humanities and Arts, University of California, Merced; Health Sciences Research Institute, University of California, Merced, Merced, California; Community Medical Centers, Fresno, California; UCSF Fresno, Fresno, California

## Abstract

**Background:**

Cryptococcal meningoencephalitis (CM) remains a devastating infection in immunocompromised patients. Diagnosis is made by using cerebrospinal fluid (CSF) and serum cryptococcal antigen (CrAg) detection by lateral flow assay (LFA), fungal cultures and more recently a multiplex polymerase chain reaction (PCR) panel. The BioFire® FilmArray® meningitis/encephalitis (FA-ME) multiplex PCR assay targets 14 pathogens and is a quick tool for evaluating patients with meningitis/encephalitis (ME). However, false negative FA-ME results have been reported in patients with CM. We performed a retrospective analysis to study the performance of the FA-ME PCR assay in comparison to other available methods in the diagnosis of CM.

**Methods:**

We identified patients with CM in our center between 2017 and 2022 using ICD9 and 10 codes (Figure 1). Data pertinent to demographics, clinical features, diagnostics of CM was collected. We included only the first spinal fluid test results that were done as part of diagnostic workup for ME for the analysis.

**Figure:**
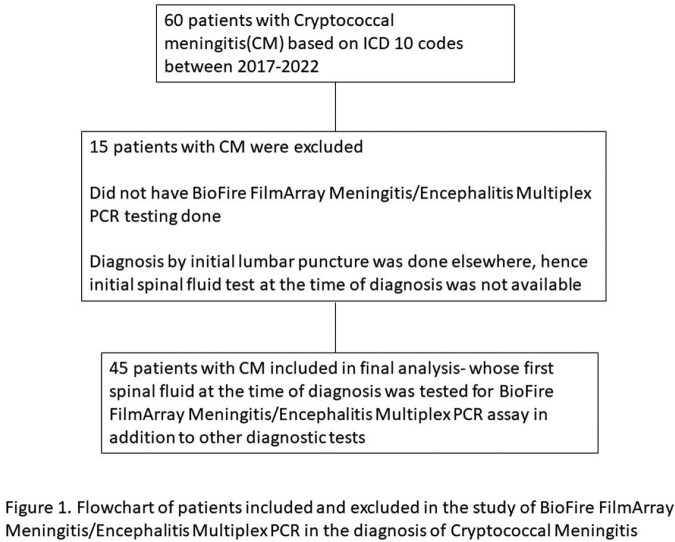

Flowchart of patients included and excluded in the study of BioFire FilmArray Meningitis/Encephalitis Multiplex PCR in the diagnosis of Cryptococcal Meningitis

**Results:**

Forty-five patients with CM met the inclusion criteria (Table 1). Of these, 12/45 had discordant false negative FA-ME multiplex PCR. Seven of the 12 patients tested falsely negative during their first episode of CM and four of them were on prior antifungals (Table 2). Thirty-six of 45 (80%) reported this was their first episode of CM. Of those, 19.44% (7 of 36) had false negative FA-ME multiplex PCR. This was found to be significantly different than the percentage with false negative FA-ME multiplex PCR in the recurrence/relapse group (55.56%; 5 of 9) using Fisher’s Exact Test (p = 0.04275). Using the Wilcoxon rank-sum test, the median of the fungal burden is significantly different between the concordant and discordant cases with the median CSF Ag titer lower in patients with false negative FA-ME results (p = 0.0016).

**Figure:**
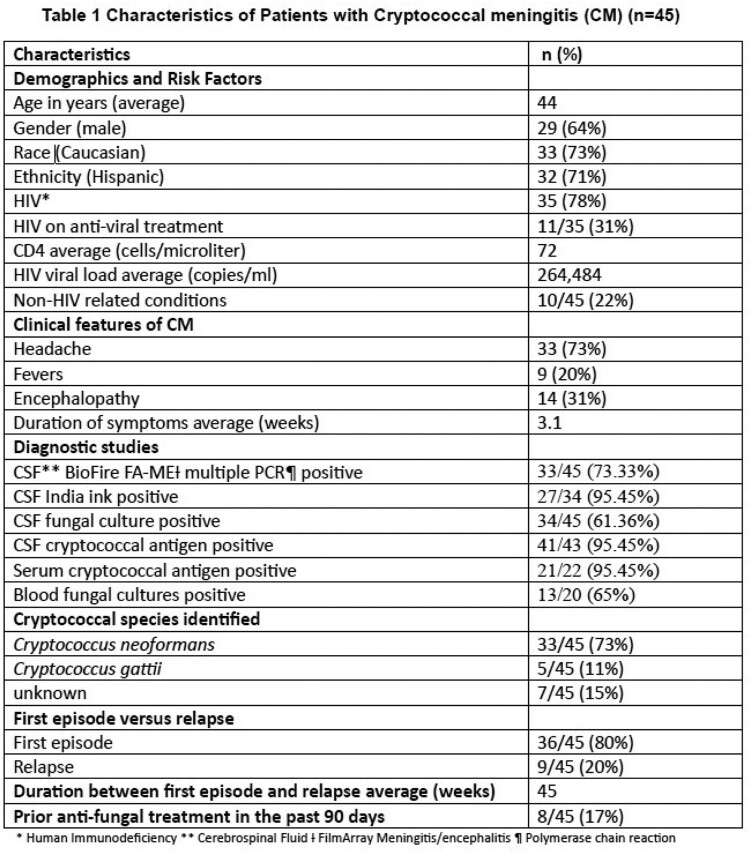

**Figure:**
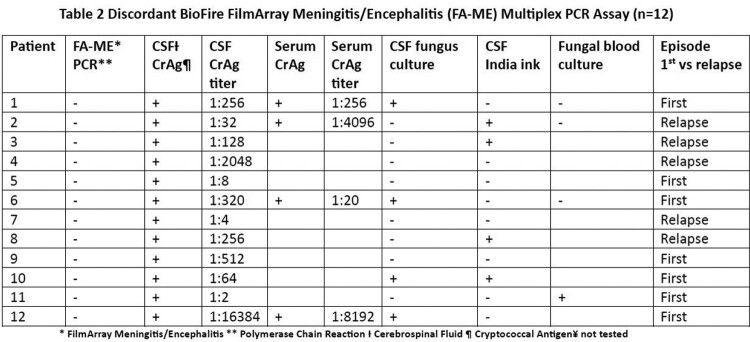

**Conclusion:**

BioFire FA-ME multiplex assay may be falsely negative in patients with cryptococcal meningitis even during their first episode. Although the chances of false negative tests are higher with lower fungal burden and in cases of relapses, caution must be used in interpreting the results. Serum CrAg was underutilized as a diagnostic aid in our patients and should be considered along with cultures and CSF CrAg when lumbar puncture is delayed.

**Disclosures:**

**All Authors**: No reported disclosures

